# Risk stratification of adult emergency department syncope patients to predict short-term serious outcomes after discharge (RiSEDS) study

**DOI:** 10.1186/1471-227X-14-8

**Published:** 2014-03-14

**Authors:** Venkatesh Thiruganasambandamoorthy, Ian G Stiell, Marco LA Sivilotti, Heather Murray, Brian H Rowe, Eddy Lang, Andrew McRae, Robert Sheldon, George A Wells

**Affiliations:** 1 Department of Emergency Medicine, University of Ottawa, Ottawa, Canada; 2 Ottawa Hospital Research Institute, Clinical Epidemiology Unit, The Ottawa Hospital, Civic Campus, 1053 Carling Avenue, 6th Floor, Rm F650, Ottawa, Ontario K1Y 4E9, Canada; 3 Department of Epidemiology and Community Medicine, University of Ottawa, Ottawa, Ontario, Canada; 4 Department of Emergency Medicine, Queen’s University, Kingston, Ontario, Canada; 5 Department of Biomedical and Molecular Sciences, Queen’s University, Kingston, Ontario, Canada; 6 Department of Public Health Sciences, Queen’s University, Kingston, Ontario, Canada; 7 Department of Emergency Medicine and School of Public Health, University of Alberta, Edmonton, Alberta, Canada; 8 Department of Emergency Medicine, University of Calgary, Calgary, Alberta, Canada; 9 Department of Medicine, University of Alberta, Calgary, Alberta, Canada

**Keywords:** Syncope, Serious outcomes, Prognosis, Arrhythmia, Emergency department, Management

## Abstract

**Background:**

While Canadian ED physicians discharge most syncope patients with no specific further follow-up, approximately 5% will suffer serious outcomes after ED discharge. The goal of this study is to prospectively identify risk factors and to derive a clinical decision tool to accurately predict those at risk for serious outcomes after ED discharge within 30 days.

**Methods/Design:**

We will conduct a prospective cohort study at 6 Canadian EDs to include adults with syncope and exclude patients with loss of consciousness > 5 minutes, mental status changes from baseline, obvious witnessed seizure, or head trauma prior to syncope. Emergency physicians will collect standardized clinical variables including historical features, physical findings, and results of immediately available tests (blood, ECG, and ED cardiac monitoring) prior to ED discharge/hospital admission. A second emergency physician will evaluate approximately 10% of study patients for interobserver agreement calculation of predictor variables. The primary outcome will be a composite serious outcome occurring within 30 days of ED discharge and includes three distinct categories: serious adverse events (death, arrhythmia); identification of serious underlying disease (structural heart disease, aortic dissection, pulmonary embolism, severe pulmonary hypertension, subarachnoid hemorrhage, significant hemorrhage, myocardial infarction); or procedures to treat the cause of syncope. The secondary outcome will be any of the above serious outcomes either suspected or those occurring in the ED. A blinded Adjudication Committee will confirm all serious outcomes. Univariate analysis will be performed to compare the predictor variables in patients with and without primary outcome. Variables with p-values <0.2 and kappa values ≥0.60 will be selected for stepwise logistic regression to identify the risk factors and to develop the clinical decision tool. We will enroll 5,000 patients (with 125 positive for primary outcome) for robust identification of risk factors and clinical decision tool development.

**Discussion:**

Once successfully developed, this tool will accurately risk-stratify adult syncope patients; however, validation and implementation will still be required. This program of research should lead to standardized care of syncope patients, and improve patient safety.

## Background

Syncope is defined as a sudden transient loss of consciousness followed by spontaneous complete recovery [1]. In pre-syncope, the patient recovers before losing consciousness [1]. Each year, it is estimated that approximately 100,000 syncope visits occur in Canadian EDs accounting for 1% of ED visits [2]. Studies from other countries report that syncope accounts for 1-3% of all ED visits [3-5]. Syncope can be caused by a spectrum of conditions ranging from the benign vasovagal syncope to life-threatening conditions (e.g. leaking abdominal aortic aneurysm or ventricular arrhythmia) that can lead to death and morbidity [6,7]. These life-threatening conditions can be classified into two distinct categories: ones that are not evident during ED evaluation (arrhythmias, and the risk of death) that need to be predicted, and serious underlying conditions that are present during ED evaluation (e.g. myocardial infarction, serious structural heart disease, significant hemorrhage, pulmonary embolism) that need to be detected. Among ED syncope patients, 7-23% will suffer serious outcomes within 7–30 days of their visit with approximately half suffering serious outcomes after ED disposition either inside or outside the hospital [7-11]. Our previous Canadian research suggests, with the present practice pattern, two-thirds of deaths and 30% of all serious outcomes that occur after ED discharge, will occur outside the hospital [2].

The decision to admit patients for evaluation or to perform a diagnostic workup in the ED are important issues as physicians need to balance the potential for serious outcomes with the reality of ED overcrowding and a shortage of in-patient hospital beds. Small pilot studies report that the yield of diagnostic tests is low and hospitalization does not improve outcomes [12,13]. Risk-stratification and disposition of syncope patients is challenging for emergency physicians as valid and reliable evidence guiding these decisions is lacking [14,15]. A clinical decision/risk stratification tool for syncope can help standardize patient evaluation, and may safely and cost-effectively assist clinicians with disposition decision.

Clinical decision tools are derived from original research that incorporates variables from history, physical examination or simple diagnostic tests to either classify patients at risk or to quantify the risk of developing serious outcomes. A syncope clinical decision tool that has undergone all three major stages of development (derivation, validation and implementation), does not currently exist [14,15].

The goal of this study is to prospectively identify risk factors and to derive a clinical decision tool for risk-stratification of adult ED syncope patients at risk for serious outcomes within 30 days of ED discharge.

### Etiology and prognosis of syncope

Syncope is caused by transient global cerebral hypoperfusion either due to decreased cardiac output or excessive vasodilatation or, more commonly, a combination of both [1]. The causes include: a) reflex (also known as vasovagal) syncope; b) cardiac syncope; c) orthostatic hypotension; and d) medications (Additional file 1) [1]. Cardiac syncope is an independent predictor of mortality and sudden death (24% in cardiac versus 3-4% in non-cardiac groups), and patients with advanced heart failure (ejection fraction ≤ 20%) have higher risk of sudden death at one-year [16-21]. A significant proportion of patients (13%-59%) will have no cause identified during their ED visit [2,6,11,16,17,22,23]. Given that high-risk patients have a mortality rate of 57% within the first year, a significant proportion of patients are admitted in the US (51% - 83%) [24-26]. However, a majority (>85%) of the patients are discharged home in Canada [8]. The decision to admit is complex and largely based on physician judgment, experience and risk tolerance. The evaluation, investigations ordered and admission rates are highly variable among ED physicians, and vary by institution and country [3,7-10,18,25-30].

### Methodological standards for clinical decision tools

Clinical decision tools are developed to reduce the uncertainty in medical decision-making [31-34]. Reported methodological standards for the development and validation of decision tools can be summarized as follows: [35-37] i) There must be a need for a decision tool because of the prevalence of the clinical condition and variability in current practice. Such a need must be a belief among physicians practicing in that area [38]; ii) The outcome or diagnosis to be predicted must be clearly defined. To reduce the risk of bias, outcome ascertainment should be made without knowledge of the predictor variables; iii) The clinical findings to be used as predictors must be clearly defined, standardized, and clinically sensible and their assessment must be done without the knowledge of the outcome (Blinded predictor variable assessment); iv) The reliability or reproducibility of the predictor variables must be clearly demonstrated; v) To increase generalizability, the subjects in the study should be selected without bias and should represent a wide spectrum of patients with and without the outcome; vi) The mathematical techniques for deriving the tools must be clearly explained; vii) Decision tools should be clinically sensible: have a clear purpose, demonstrate content validity, must be relevant, concise and easy to use in the intended clinical context; viii) The accuracy of the decision tool in classifying patients with (sensitivity) and without (specificity) the targeted outcome should be demonstrated; ix) Prospective validation on a new set of patients is an essential step in the evolution of this form of decision support. Unfortunately, many clinical decision tools are not prospectively validated to determine their accuracy, reliability, clinical sensibility, or potential impact on practice. This validation process is very important because many statistically-derived tools fail to perform well when tested in a new population. The reason for this poor performance may be statistical (i.e., overfitting or instability of the original derived model) or due to differences in prevalence of disease or differences in the population or differences in how the decision tool is applied [39-41]; x) An implementation phase (to demonstrate the true effect on patient care) is the ultimate test for a decision tool in terms of effectiveness, uptake and cost [42].

### Previous emergency department syncope studies

There are nine original studies previously published to predict SAEs in ED syncope patients [7,10,11,24,43-47]. A synopsis of the available instruments and how they perform against the above-mentioned methodological standards is given in Table 1. All published studies define ‘abnormal ECG’ variable differently and none are based on evidence. The definitions for ‘abnormal ECG’ in published studies are detailed in “Definition of Abnormal Electrocardiogram (ECG) in the Syncope Risk-Stratification Studies”. All published studies also include patients with serious outcomes during the ED evaluation in the derivation cohort. Inclusion of such patients in tool derivation biases the tool towards the identification of patients with obvious serious outcomes and leads to poor performance on external validation. In summary, there are very few prospective studies that assess for all short-term serious outcomes; however, all have poor diagnostic test characteristics and several methodological flaws that preclude widespread use [7,11,45]. Hence, there is no well-validated clinical decision tool that exists to help physicians standardize evaluation of ED syncope patients and identify those at risk for serious outcomes within 30 days. We plan to derive a robust tool without the above listed weaknesses.

**Table 1:** Emergency department syncope studies

**No**	**Year**	**Study**	**Variables**	**Scoring system**	**Endpoints**	**Results**^**1**^	**Strengths**	**Weakness**
1	1997	Martin et al.	• Abnormal ECG	0 to 4	1-year arrhythmias or deaths	4.4% score 0	One of the earliest studies	Only long-term outcomes
			• History of ventricular arrhythmia	(1 point for each item)				
			• History of CHF			57.6% score 3 or 4		Not validated
			• Age >45 years					
2	2002	OESIL	• Abnormal ECG	0 to 4	1-year mortality	0% score 0	Externally validated for	Only long-term outcomes
			• History of cardiovascular disease	(1 point for each item)		0.6% score		
			• Lack of prodrome			14% score 2	up to 6 month outcomes	Modest performance for outcomes up to 6 months
			• Age >65 years					
						29% score 3		
						53% score 4		
3	2003	Sarasin et al.	• Age >65 years	0 to 3	Arrhythmias in unexplained ED syncope	2% score 0	Studied arrhythmia risk in unexplained syncope	Only inpatients
			• History of CHF	(1 point for each item)		17% score 1		Internal validation on historical cohort
			Abnormal ECG			35% score 2		
						27% score 3		
								No external validation
4	2004	San Francisco Syncope Rule	• Abnormal ECG	No item = No risk	7-day serious events	Sensitivity 98%	First tool for short-term events	Wide variations in performance
			• History of CHF					
			• Shortness of breath					
			• Hematocrit < 30%	≥ 1 item = risk		Specificity 56%	Most widely validated	ECG variable too broad
			• Triage systolic BP <90 mmHg					
								Included soft outcomes^2^
5	2007	Boston Syncope Rule	• Compilation of 25 plausible variables	≥ 1 item = risk	30-day serious events	Sensitivity 97%	A thorough list of variables	No statistical methods
						Specificity 62%		Not practical
								No external validation
6	2008	STePS	• Abnormal ECG	≥ 1 item = risk	10-day and 1-year events	Not Reported	Addresses the role of admissions to hospital	Readmission to hospital was an outcome
			• Trauma					
			• No prodrome					
			• Male sex					
								Not validated
7	2008	EGSYS	• Palpitations before syncope (+4)	Addition of all items	Cardiac syncope probability	2% score <3	First study to incorporate variables from history	Not generalizable - Syncope expert always available
			• Abnormal ECG and/or heart disease (+3)					
			• Syncope during effort (+3)		2-year total mortality	13% score 3		
			• Syncope while supine (+2)			33% score 4		
						77% score >4		Internal validation 92% sensitivity
						2% score <3		
						21% score ≥3		No robust external validation
			• Autonomic prodrome (−1)					
8	2009	Sun et al.	• Age >90 years (+1)	Addition of all items	30-day events among older (≥ 60 years) syncope patients	2.5% score −1, 0	First study to risk stratify older patients	Retrospective
			• Male sex (+1)					
			• History of arrhythmia (+1)			6.3% score 1,2		Can be applied only to older patients
			• Triage systolic BP >160 (+1)				Large sample size	
			• Abnormal ECG (+1)					
			• Abnormal troponin I (+1)			20% score 3 to 6		Not validated
			• Near-syncope (−1)					
9	2010	ROSE	• BNP level ≥300 pg/ml	Presence of any item	1-month serious events	Sensitivity 87%	First study to evaluate the role of BNP in risk stratification	Short-term events included stroke
			• Bradycardia ≤50 in ED/pre-hospital					
			• Positive fecal occult blood on rectal			Specificity 66%		
			• Anemia – Hemoglobin ≤ 90 g/L					Requires BNP testing that is not widely available
			• Chest pain with syncope					
			• Q wave on ECG (except in lead III)					
			• O_2_ saturation ≤ 94% on room air					Less than ideal sensitivity

ECG = Electrocardiogram, CHF = Congestive Heart Failure, OESIL = Osservatorio Epidemiologico sulla Sincope nel Lazio, BP = Blood Pressure, STePS = Short-Term Prognosis of Syncope, EGSYS = Evaluation of Guidelines in Syncope Study, BNP = Brain type or B-type Natriuretic Peptide.

^1^Results of validation phase when available.

^2^Soft outcomes = Cortical stroke and hospitalization on return visit with no serious events.

All studies used standard statistical methods to develop the tool except the Boston Syncope Rule study.

### Definition of Abnormal Electrocardiogram (ECG) in the Syncope Risk-Stratification Studies

This section details the variations in the definition of the ‘abnormal ECG’ variable in the different studies.

**Martin et al. **[17]**:** Abnormal ECG is defined as presence of any of the following: atrial fibrillation or flutter, multifocal atrial tachycardia, junctional or paced rhythms, frequent or repetitive runs of premature ventricular contractions or ventricular tachycardia, left axis deviation, bundle branch block, intraventricular conduction delay, left or right ventricular hypertrophy, PR interval < 10 seconds, previous myocardial infarction, II or III degree atrioventricular block. Isolated sinus bradycardia or sinus tachycardia and non-specific ST-T wave abnormalities were considered normal.

**OESIL (Osservatorio Epidemiologico sulla Sincope nel Lazio) study:** The following ECG abnormalities were considered abnormal in the OESIL study: 1) Rhythm abnormalities: Supraventricular tachycardia, multifocal atrial tachycardia, atrial fibrillation, frequent or repetitive premature supraventricular or ventricular complexes, sustained or non-sustained ventricular tachycardia or paced rhythms; 2) Conduction disorders: Complete or Mobitz type I or type II atrioventricular blocks, bundle branch block or intraventricular conduction delay; 3) Ventricular hypertrophy right or left; 4) Left axis deviation; 5) Old myocardial infarction; 6) Myocardial ischemia: ST segment and T wave abnormalities consistent or possible with myocardial ischemia. Non-specific repolarization abnormalities were considered normal.

**Sarasin et al. **[44]**:** The ECG was considered abnormal if any one of the following abnormalities were present: atrial fibrillation, sinus pause ≥ 2 and < 3 seconds, sinus bradycardia > 35 and ≤ 45 beats per minute, conduction abnormalities (bundle branch block, second-degree Mobitz type I atrioventricular block, bifascicular block), signs of previous myocardial infarction or ventricular hypertrophy or multiple premature ventricular beats were present.

Abnormalities worse than the above were considered diagnostic of the cause of syncope and included: sinus pause ≥ 3 seconds, sinus bradycardia ≤ 35 beats per minute, atrial fibrillation with slow ventricular response defined as RR interval ≥ 3 seconds, Mobitz type II atrioventricular block, complete atrioventricular block or symptomatic or sustained ≥ 30 seconds ventricular tachycardia. Minor abnormalities such as I degree atrioventricular block, non-specific ST-T wave abnormalities, sinus tachycardia and premature atrial contractions were considered normal.

**San Francisco Syncope Rule Study:** Presence of non-sinus rhythm and any new changes in comparison to the previous electrocardiogram was considered abnormal. If no old electrocardiogram is available then any changes present are sufficient to classify the electrocardiogram as abnormal.

**STePS (Short-Term Prognosis in Syncope) Study:** ECG was defined as abnormal if any of the following were present: 1) atrial fibrillation or tachycardia; 2) sinus pause ≥2 seconds; 3) sinus bradycardia with heart rate ranging between 35 and 45 beats/min; 4) conduction disorders (i.e., bundle branch block, second-degree Mobitz I atrioventricular block); 5) ECG signs of previous myocardial infarction or ventricular hypertrophy; and 6) multiple premature ventricular beats.

**EGSYS (Evaluation of Guidelines in Syncope Study):** The abnormalities that classified the ECG as abnormal in the EGSYS study were: Sinus bradycardia, atrioventricular block greater than first degree, bundle branch block, acute or old myocardial infarction, supraventricular or ventricular tachycardia, left or right ventricular hypertrophy, ventricular pre-excitation, long QT and Brugada pattern.

**Sun et al. **[47]: ECG was considered abnormal if any of the following were present: non-sinus rhythm, sinus rhythm with pulse rate < 40 beats/min, Q/ST/T changes consistent with acute or chronic ischemia, abnormal conduction intervals (QRS >0.1 milliseconds, QTc >450 milliseconds), left or right ventricular hypertrophy, left axis deviation, and bundle branch block.

### Professional society guidelines

There are also three pertinent clinical guidelines from professional societies for risk stratification of ED syncope patients [1,14,48]. The European Society of Cardiology published guidelines for admission in 2001, 2004 and recently updated them in 2009 (European Society of Cardiology - Guidelines for Admission of Syncope Patients) [1,49,50]. Studies validating these guidelines either found no effect or were of poor methodological quality [51,52].

### European Society of Cardiology - Guidelines for Admission of Syncope Patients

The European Society of Cardiology **2001 and 2004 guidelines** for admission are similar and recommend admission either for diagnosis or treatment and are as follows:

1. For diagnosis

• Suspected or Known significant heart disease,

• Abnormal ECG – Defined by the presence of any one of the following abnormalities: Bifascicular block (left bundle branch block or right bundle branch block combined with left anterior or left posterior fascicular block), QRS duration ≥ 0 · 12 seconds, Mobitz I second degree atrioventricular block, Asymptomatic sinus bradycardia (<50 beats/min) or sinoatrial block, Pre-excited QRS complexes, Prolonged QT interval, Right bundle branch block pattern with ST-elevation in leads V1-V3 (Brugada syndrome), Negative T waves in right precordial leads, epsilon waves and ventricular late potentials suggestive of arrhythmogenic right ventricular dysplasia or Q waves suggesting myocardial infarction,

• Syncope during exercise or supine,

• Associated severe injury,

• Family history of sudden death,

• Sudden preceding palpitations before syncope,

• Frequent recurrent episodes, or high suspicion of cardiac syncope.

2. For treatment if the following conditions are the cause

• Cardiac arrhythmias,

• Cardiac ischemia,

• Structural cardiac or cardiopulmonary disease,

• Stroke or focal neurological disorders, or

• For pacemaker insertion.

The **2009 guidelines** added non-sustained ventricular tachycardia and severe co-morbidities (severe anemia and electrolyte disturbances) to the admission criteria.

The American College of Emergency Physicians issued guidelines for management of ED syncope patients in **2001 and 2007**[48,53]. The 2001 guidelines recommend admission if any of the following high-risk features is present: 1) History of congestive heart failure or ventricular arrhythmias, 2) Presence of chest pain or acute coronary syndrome, 3) Signs of heart failure or valvular heart disease, or 4) ECG signs of ischemia, arrhythmia, prolonged QT interval, or bundle branch block. The guidelines recommend that hospitalization be considered if any of the following medium-risk features are present: 1) Age >60 years, 2) Abnormal ECG (defined as changes consistent with acute ischemia, dysrhythmias, or significant conduction abnormalities), 3) Family history of sudden death, or 4) Young patients with unexplained exertional syncope. One study validated the **2001 guidelines** retrospectively but outcomes were limited to cardiac syncope with serious methodological limitations in attributing the cause of syncope as cardiac [54]. The 2007 guidelines advise hospitalization if any of the following features are present: 1) Older age with associated comorbidities, 2) Abnormal ECG (defined as changes consistent with acute ischemia, dysrhythmias, or significant conduction abnormalities), 3) Hematocrit <0.3, or 4) History or presence of congestive heart failure or coronary or structural heart disease. The 2007 guidelines included variables ‘older age with associated comorbidities’ and ‘abnormal ECG’ that were not clearly defined and these guidelines have not been validated.

The Canadian Cardiovascular Society published a position paper on the standardized approaches to the management of syncope and identified major and minor risk factors for short-term events [14]. These risk factors have not been validated yet. The guideline recommends urgent cardiac assessment within 2 weeks if any one of the following major risk factors are present: 1) Abnormal electrocardiogram (any bradyarrhythmias, tachyarrhythmia or conduction disease), 2) History of cardiac disease (ischemic, arrhythmic, obstructive, valvular), 3) Hypotension (systolic blood pressure <90mmHg), or 4) Heart failure (past or current state). The guideline also recommends that an urgent cardiac assessment be considered if any one of the minor factors is present: 1) Age >60 years, 2) Dyspnea, 3) Anemia (haematocrit <0.30), 4) hypertension, 5) cerebrovascular disease, 6) family history of sudden death (age <50 years, or syncope during special situations (while supine, exercise or with no prodrome).

### Objectives

The overall goal of this study is to prospectively identify risk factors and to derive a clinical decision tool for risk-stratification of adult ED syncope patients to accurately predict those at risk for serious outcomes within 30 days of ED discharge. Specific objectives include:

i. To develop and test standardized clinical assessments in adult ED syncope patients.

ii. To collect patient characteristics, historical data, physical examination details, specific ECG characteristics, duration of cardiac monitoring, cardiac monitor abnormalities and results of investigations conducted.

iii. To determine the inter-observer reliability for the clinical information collected.

iv. To collect data regarding the time of occurrence of all serious outcomes within 30 days (in the ED, as inpatient or outside the hospital).

v. To determine the statistical association between clinical information and occurrence of serious outcomes within 30 days but after ED discharge.

vi. To use multivariate analysis to identify ECG abnormalities that is associated with cardiovascular serious outcomes.

vii. To assess the role of B-type or Brain type Natriuretic Peptide (BNP) in a subset of patients.

viii. To use multivariate analysis to identify risk factors associated with serious outcomes within 30 days of ED discharge.

ix. To develop a highly sensitive and adequately specific clinical decision tool to guide disposition decision-making.

x. To use multivariate analysis and develop specific criteria to identify syncope patients who need cardiac monitoring in the ED and the optimal duration of such monitoring to avoid missing serious outcomes that occur within a few hours.

xi. To assess the potential impact on resource utilization due to the newly developed clinical decision tool for 30-day serious outcomes.

xii. To determine emergency physicians’ accuracy in predicting 30-day serious outcomes.

xiii. To assess for long-term outcomes among ED syncope patients and develop guidelines for appropriate follow-up.

## Methods

### Study design and setting

We will conduct a prospective observational cohort study to enroll ED syncope patients. The study setting will be six Canadian teaching hospitals with a combined annual ED volume of approximately 300,000 patient visits (Ottawa Hospital Civic and General Campuses - Ottawa, ON; Kingston General Hospital and Hotel Dieu Hospital - Kingston, ON; Foothills Medical Centre – Calgary, AB; University of Alberta Hospital – Edmonton, AB).

### Study population

We will include adult ED patients with syncope (sudden, transient loss of consciousness followed by spontaneous, complete recovery) and exclude those with prolonged loss of consciousness (>5 minutes), mental status changes from baseline, witnessed obvious seizure, significant trauma requiring admission and those with loss of consciousness due to alcohol intoxication, illicit drug abuse, or secondary to head trauma.

### Patient enrolment

On duty ED physicians or research assistants will screen consecutive patients presenting with syncope, pre-syncope, fainting, black out, loss of consciousness, fall, collapse, seizure, dizziness or light-headedness. ED physicians or research assistants will apply the above-mentioned inclusion and exclusion criteria on these patients to confirm their eligibility. We will include patients only once in the study to avoid double counting. All patients’ assessments will be made by staff physicians certified in emergency medicine by the Royal College of Physicians and Surgeons of Canada and/or the College of Family Physicians of Canada or emergency medicine residents. Standardized description of all variables and outcomes will be appended to the data collection form. Our research team will also orient physician assessors to the components of the standardized assessment and definitions of the variables, by regular presentations and group sessions. Physicians will be asked to fill the data collection form immediately after their initial history and physical examination, and will be requested to complete the rest of the form when results of investigations (blood tests, ECG) that are deemed necessary as per the treating physician are available. Results of our retrospective phase indicate that a small proportion of patients do not have blood tests (11%) or an ECG (7%) performed as part of the ED work-up [2]. While there is no convincing evidence, guidelines from professional organizations recommend but do not mandate ECG on all syncope patients [1,14]. Published studies report that blood tests are helpful only in a small proportion (2-3%) of syncope patients [16-18]. As there is lack of strong evidence for performing both ECG and blood tests on all syncope patients and as the study protocol does not alter current practice, we believe ethically we cannot mandate these tests be performed.

### Selection of variables

The variables selected for collection in this study were chosen based on: 1) A recently concluded comprehensive literature search done as part of developing a position statement for the Canadian Cardiovascular Society; 2) Recommendations by a committee of three cardiologists with decades of syncope research experience, eight experienced emergency physicians and three methodology experts; and 3) The results of our previously completed studies [14,55,56]. Consideration was given to restricting the variable list as it may affect enrollment efficiency and time required to complete the physician data collection form. The variables that will be collected in this study are provided in Prospective Multicentre ED Syncope Study: List of Variables Collected and their Definitions.

Prospective Multicentre ED Syncope Study: List of Variables Collected and their Definitions

1. **Variables from History: *a) Demographics*** - age, sex; ***b) Details of the event –*** was it witnessed, any predisposing factors, position during the episode, exertion prior to syncope, occurrence and duration of prodromal symptoms, palpitations prior to syncope, orthostatic symptoms, any associated symptoms, any injuries suffered; ***c) Past Medical History*** - atrial or ventricular arrhythmias, congestive heart failure, coronary or valvular heart disease, cardiomyopathy, pacemaker or implantable cardioverter-defibrillator insertion, renal failure, hypertension, diabetes, stroke, transient ischemic attack, gastrointestinal bleeding, pulmonary hypertension, pulmonary embolism, deep venous thrombosis, peripheral arterial disease, seizure, syncope, malignancy, other cardiac conditions (cardiac tumors, pericardial disease, congenital coronary abnormalities, prosthetic valve dysfunction, myocarditis); ***d) Personal or Family history*** of congenital heart disease, prolonged QT, Brugada syndrome; ***e) Family history*** of sudden deaths; or ***f) Medications*** – exogenous estrogens.

2. **Variables from Pre-hospital: *a) Arrival by ambulance***; and ***b) Paramedic findings*** - first and the lowest systolic and diastolic blood pressure (BP), non-sinus rhythm or arrhythmia detected on ambulance rhythm strip/cardiac monitor, symptoms such as light-headedness/dizziness, syncope/pre-syncope, or hypotension defined as systolic BP < 90 mmHg associated with rhythm abnormalities; or any cause for syncope found by paramedics.

3. **Variables from Physical Examination: *a) Triage vital signs*** - pulse rate, systolic and diastolic BPs, respiratory rate, oxygen saturation; ***b) Postural systolic and diastolic BP*** – lying and after 3 minutes of sitting or standing if orthostatic symptoms present; ***c) Lowest and highest*** systolic and diastolic BP, and heart rate recorded; ***d) Glasgow Coma Scale***, score based on eye opening, verbal and motor response; and ***e) Examination findings*** - presence of murmur, congestive heart failure, clinical signs of deep venous thrombosis, tenderness in the abdomen, and presence of bright red blood per rectum or stool occult blood.

4. **Variables from Investigations: *a) Laboratory values*** - hemoglobin, hematocrit, sodium, potassium, chloride, glucose, urea, creatinine, creatine kinase, troponin and Brain Natriuretic Peptide (BNP). If several values are available we will choose the lowest values of hemoglobin and hematocrit, most extreme values of sodium and potassium, and highest values of urea, creatinine, creatine kinase and troponin. We will also collect the last available values for hematocrit and creatinine; ***b) Radiological investigations*** – abnormalities on chest x-ray, computerized tomography of the head and results of previous available echocardiogram. ***c) ECG*** - rate, rhythm, axis, QRS duration, PR and corrected QT interval, and presence of any of the following: premature atrial or ventricular beats, blocks, ventricular hypertrophy, old myocardial infarction, new ischemic changes, or others (delta waves, changes consistent with Brugada syndrome, arrhythmogenic right ventricular dysplasia); and ***c) Cardiac monitor***: Duration of monitoring, arrhythmia detected and symptoms associated with the arrhythmia.

5. **Variables for Disposition and Physician Judgment: *a) Final diagnosis in ED*** - vasovagal, orthostatic hypotension, likely cardiac, cause unknown, or outcome in the ED or pre-hospital setting; ***b) Certainty of diagnosis*** in ED - scaled from 0 to 100% by the deciles; ***c) Probability of SAE*** occurring within 30 days after leaving the ED - scaled from 0-5% by each percentile, 10-50% by the deciles, 75% and 100%; ***d) Referral:*** Specialty to which patient was referred (e.g., cardiology, or neurology) and when (in ED or as outpatient), disposition of the patient; ***e) Outpatient investigations*** organized – holter, echocardiogram or others; and ***e) Definitive Treatment*** – Did the patient/family decline definitive treatment?

### Interobserver reliability

Whenever possible, a second emergency physician blinded to the findings of the first assessor will collect variables on the physician data collection form approximately in 10% of the study patients. This second physician will obtain consent from the patient and will explicitly inform the patient that the assessment is being done purely for research purposes.

### Quality assurance

Feedback will be given to physicians regarding any issues with their data collection or recording. We will not provide physicians with information regarding the accuracy or reliability of the individual variables.

### Ethical considerations

We have obtained ethics approval from the research ethics boards of all the study hospitals. All personal identifiers will be kept strictly confidential and stored separately from the clinical information collected. We will also explicitly advise physicians not to alter their patient care for the study, but to continue with usual care. As there is no change in patient management, this study poses no threat to patient safety.

This study is approved by the Research Ethics Boards of all the study hospitals.

### Outcomes

The primary, secondary and long-tem (1-year) outcome measures are detailed in Prospective Multicentre ED Syncope Study: List of Serious Adverse Events and their Definitions. We will assess for outcome occurrence by a three step approach - first we will use the computerized hospital patient-tracking system to review the following records for outcome occurrence at all local adult hospitals: a) ED and inpatient hospital records of return visits; b) Autopsy reports; c) Outpatient clinical notes; and d) Results of outpatient investigations. As a second step we will conduct a 30 day and 1-year telephone follow-up (with a verbal consent obtained again at the time of telephone contact) to verify that the patient is alive and has not sustained any serious outcome. During telephone follow-up, we will ask questions regarding ED or outpatient clinic visits after the index event, any new diagnosis or procedural interventions, and if the cause for the syncope has been identified. If a patient refuses telephone follow-up, no further action will be taken and he/she will be designated as lost to follow-up.

### Prospective Multicentre ED Syncope Study: List of Serious Adverse Events and their Definitions

The **primary outcome** will be a serious adverse event (SAE) occurring after ED disposition and within 30 days of the index event, either inside or outside hospital. SAE is defined as any one of the following: death, arrhythmia, myocardial infarction, serious structural heart disease, aortic dissection, pulmonary embolism, severe pulmonary hypertension, subarachnoid hemorrhage, significant hemorrhage or procedural interventions to treat a cause of syncope. The definitions of the serious adverse events are detailed below:

a) ***Death:*** due to a cause of syncope or unknown causes;

b) ***Arrhythmias:*** Sustained (> 30 seconds) or polymorphic ventricular tachycardia; sinus bradycardia < 40 beats/minute; sick sinus with alternating sinus bradycardia and tachycardia; sinus pause > 3 seconds; Mobitz type II atrioventricular heart block; complete heart block or junctional/idioventricular rhythm; alternating left and right bundle branch block; symptomatic (light-headedness/dizziness, hypotension – systolic BP < 90 mmHg) supraventricular tachycardia with rate > 100/minute; symptomatic atrial flutter or fibrillation with fast (>100/minute) or slow (RR interval > 3 seconds) ventricular rate; pacemaker or implantable cardioverter-defibrillator (ICD) malfunction with cardiac pauses, or an abnormal electrophysiological study (corrected sinus node recovery time > 550 milliseconds; His-Ventricular intervals >100 milliseconds; inducible ventricular tachycardia for > 30 second; polymorphic ventricular tachycardia/ventricular fibrillation in patients with Brugada or ventricular dysplasia or previous cardiac arrest; symptomatic supraventricular tachycardia, or Infra-Hisian block);

c) ***Myocardial infarction:*** Defined as a clinically important elevation in troponin or ECG change and must have been confirmed by the emergency physician or cardiologist or the most responsible physician;

d) ***Identification of serious structural heart disease***: i) aortic stenosis with valve area ≤ 1 cm^2^; ii) hypertrophic cardiomyopathy with outflow tract obstruction; iii) left atrial myxoma or thrombus with outflow tract obstruction; or iv) pericardial effusion with ventricular wall motion abnormalities or pericardial tamponade;

e) ***Aortic dissection*** - confirmed by CT chest, trans-esophageal echocardiogram, MRI or angiography;

f) ***Pulmonary embolism*** – confirmed by VQ scan, computed tomography scan of the chest or angiography and the patient should have received or should have been considered for treatment;

g) ***Identification of severe pulmonary artery hypertension*** – detected by cardiac catheterization or echocardiography with a mean pulmonary arterial pressure > 30 mmHg;

h) ***Subarachnoid hemorrhage*** - Confirmed by computed tomography/magnetic resonance imaging of the brain with or without spinal fluid analysis by lumbar puncture;

i) ***Significant hemorrhage*** - Defined as syncope associated with detected source of bleeding such as gastrointestinal bleeding, ruptured abdominal aortic aneurysm, or ectopic pregnancy that is clinically significant to cause syncope in the opinion of the treating physician or that required transfusion;

j) ***Procedural interventions -*** Any interventions used to treat a cause of syncope. The procedural interventions include pacemaker and/or defibrillator insertion, cardioversion for arrhythmias, surgery for valvular heart disease, dialysis for electrolyte abnormalities causing arrhythmia, chest tube/pig tail catheter insertion for pneumothorax or pleural effusion, or surgery for abdominal aortic aneurysm or ruptured spleen.

The **secondary outcome** will be any of the above-mentioned SAE occurring in the ED.

We will also collect **long-term (1-year) outcomes**. Long-term outcomes include death, arrhythmias, serious structural heart disease, and cardiac procedural interventions for arrhythmia or structural heart disease. The definitions for the long-term outcomes are the same as the 30-day SAEs.

If patient follow-up cannot be achieved by the above-mentioned two steps, we will review the provincial health systems database and death records from the provincial coroner’s office for matching patients. Final determination of serious outcomes occurrence will be completed by an independent adjudication committee blinded to the predictor variables comprised of two ED physicians. In cases of disagreement, a third physician (ED physicians or internist or cardiologist) will adjudicate the outcome. For serious outcomes that occur or those detected after ED discharge, we will collect information if the outcome was anticipated during ED evaluation.

### Data analysis

Data will be entered into a computerized database using Statistical Analysis System (SAS Institute Inc., Cary, NC, USA) software. We will ensure accuracy of data entry with built-in logic checks and also by regular review of frequency reports. We will calculate the inter-observer agreement using the kappa coefficient for each variable in a convenience sample 10% of study patients independently assessed by a second emergency physician. We will consider individual predictor variables with kappa value ≥0.6 for multivariate analysis [57]. We will analyze the timing of occurrence of serious outcomes in ED (to calculate optimal duration patients need to be monitored in ED) and days of occurrence of outcomes after ED visit (for insight into follow-up of these patients).

### Univariate analysis

Univariate analysis will be performed to determine the strength of association between the predictor variables and serious outcomes. We will choose the appropriate univariate technique based on the type of data: chi square test with continuity correction for nominal variables; unpaired, two tailed t-test for continuous variables, using pooled or separate variance estimate as appropriate; and, the Mann–Whitney U test for ordinal variables. For continuous variables we will assess for the most discriminative cut point to include in multivariate analysis.

### Multivariate analysis

Variables that are reliable (kappa ≥0.6) and strongly associated with serious outcomes (p-value <0.2) will be selected for multivariate analysis by logistic regression using Statistical Analysis System (SAS) software or using recursive partitioning. We will use multivariate analysis to identify the risk factors for serious outcomes within 30 days of ED discharge and to derive a clinical decision tool. When building the model, for missing predictor variables, we plan to either impute by multiple imputations or assume as normal if it is in young patients with obvious vasovagal syncope [58]. We will evaluate interaction among predictor variables using Mantel-Haenszel and logistic model procedures. We will consider appropriate combining of variables and use composite variables for incorporation (e.g. bifascicular block). We will assess for multicolinearity in the model, a statistical phenomenon in which two or more predictor variables are highly correlated. We will also assess for lack of fit by Hosmer-Lemeshow goodness-of-fit test and for overfitting in the model by Akaike information criterion [59,60]. If difficulties arise in developing a clinical decision tool with acceptable diagnostic test characteristics, we will explore the option of building models with separation of serious events: before and after the 7-day mark; detection of serious underlying conditions (serious structural heart disease, aortic dissection, pulmonary embolism, severe pulmonary hypertension, subarachnoid hemorrhage or significant hemorrhage) versus prediction of serious events (arrhythmia or death); separation of cardiac versus non-cardiac events; and excluding patients with procedural interventions performed without evidence of underlying serious condition (e.g. pacemaker insertion without evidence of bradyarrhythmia). If there is considerable variation in the ED length of stay among the study patients, we will also explore the option of the primary outcome as occurrence of serious outcome 6 hours after ED arrival. The majority of syncope patients are assigned a Canadian Triage and Acuity Scale (CTAS) 3. As a result, in most Canadian provinces it is expected that a disposition decision is made within six hours of arrival. Hence, we will choose a 6 hour cut off point if needed.

We will aim for a model that is highly sensitive, adequately specific, clinically sensible and acceptable, and one that will not require any computation or statistical aids so that it can be easily incorporated into practice. We will also derive another model (with aim of 100% sensitivity) to predict patients who will benefit from cardiac monitoring while in the ED.

### Classification performance

As a preliminary validation, we will assess the performance of the tool by comparing the classification of each patient against the occurrence of serious outcome. This will allow 95% confidence interval (CI) estimation for the sensitivity and specificity of the derived tool. We will perform an internal validation of the scale across 1,000 replications using the bootstrap method [61]. A more robust validation will be carried out later.

### Resource utilization and physician judgment

We will calculate and compare the actual admission rates versus hypothetical rates if the new tool were implemented. We will calculate the proportion of patients with correct diagnosis made during the ED visit and the proportion of patients who suffer serious outcome outside the hospital with no specific follow-up arrangements. From the physician prediction probabilities and the model for the new scale, we will calculate and compare the likelihood ratios and area under the receiver operating characteristic (ROC) curves.

### Sample size

5,000 patients will be enrolled at the six study sites. Since there is no hypothesis being tested, we determined the sample size based on the number of variables in the final model and the estimation of precision of the sensitivity of the tool to be derived in the study population. Previous studies have identified that there must be at least 10 events per predictor variable in the final model [62]. For clinical decision tool studies, the specific approach taken (bound on the error of estimation which is the width of the 95% CI estimate) is the standard technique used in sample size calculation for decision tool studies [63]. Conservatively assuming the prevalence as 2.5%, we calculated a total sample size of 5,000 patients with 125 patients suffering serious outcomes within 30-days of ED discharge will be needed to derive the tool.

### Methodological issues

We considered the following methodological issues during the planning of this study.

#### Exclusion of pre-syncope patients

There is no standardized definition for the symptom ‘pre-syncope’ and published studies are contradictory with respect to the prognosis of pre-syncope in comparison to syncope [47,64-66]. One study reports that pre-syncope has a benign prognosis, [47] another reports that it is a non-specific symptom with cardiac monitor showing sinus rhythm when captured [65] while two other studies report that the prognosis is the same as syncope [64,66]. The European Society of Cardiology guidelines concluded that the pathophysiology might be different for pre-syncope than syncope [1]. Hence, we elected to exclude pre-syncope patients.

#### Inclusion of admitted patients

There are no clear guidelines for admission of syncope patients. Hence, patients are usually admitted based on subjective physician judgment. It is possible that the same patient if seen by another emergency physician could be discharged home from the ED as evident by the wide variations in admission proportions among physicians, hospitals and countries [7,8,10,25-27]. Inclusion of admitted patients will allow for more robust risk factor identification and derivation of a clinical decision tool with the highest sensitivity to predict all serious outcomes after ED disposition. This will avoid misclassification of high-risk patients as low-risk. We will however classify patients who suffer serious outcomes during hospital admission as having occurred in the ED, if their outcome was expected or suspected during ED evaluation.

#### 30 day versus 7 day outcomes

In Canada and in most western countries, there are no dedicated ‘syncope clinics’ and follow-up with an internal medicine specialist or a cardiologist is not generally possible within 7-days. Our pilot study showed that a significant proportion (37%) of the serious outcomes occurred between 7 and 30 days of the index syncope visit [2]. The patients with serious outcomes occurring within 7-days of ED visit will benefit the most from inpatient admission, while those patients who suffer serious outcomes after 7-days will benefit from expedited outpatient follow-up. Hence, we will assess for 30-day outcomes.

## Discussion

In Canada, as in many other jurisdictions, there is constant pressure to avoid hospital admission due to ED overcrowding and bed shortages. Our current practice fails to identify adult syncope patients at risk for serious outcomes not evident during ED evaluation, and consequently a small but important number of patients suffer serious outcomes after ED discharge. This study will identify risk factors associated with serious outcomes among syncope patients within 30 days of ED discharge. We will also derive a clinical decision tool to identify those syncope patients at risk for short-term SAE and require emergent testing/treatment and/or admission. Once the tool is derived, we plan to validate it in a subsequent study. Upon validation, this tool has the potential to standardize care of syncope patients including cardiac monitoring and the duration of monitoring in the ED, disposition and urgency of further investigations/treatment. The tool has the potential to prevent morbidity and mortality suffered by syncope patients outside the hospital and efficiently use in-patient resources. We strongly suspect that once the tool is derived and validated, it will be useful to ED physicians, cardiologists, internists and family physicians to risk-stratify adult syncope patients who are at risk for serious outcomes.

## Competing interests

The authors declare that they have no competing interests.

## Authors’ contributions

All authors listed on the manuscript have made substantial contributions to the conception and design of the study. GAW made contributions to the study design and data analyses plan. IGS, MS, HM, BR, EL, AMR, RS collect data at the sites. VT drafted the article; all other authors revised it critically for important or intellectual content, and approved the final version.

## Pre-publication history

The pre-publication history for this paper can be accessed here:

http://www.biomedcentral.com/1471-227X/14/8/prepub

## Supplementary Material

Additional file 1 Causes of Syncope. Click here for file
