# Supplementary material for: Risk stratification of adult emergency department syncope patients to predict short-term serious outcomes after discharge (RiSEDS) study
Source: BMC Emerg Med. 2014 Mar 14;14:8. doi: 10.1186/1471-227X-14-8 (PMC4003802; doi:10.1186/1471-227X-14-8)
Supplement: Additional file 1 — Causes of Syncope. [file 1471-227X-14-8-S1.doc]

**Additional file 1: Causes of Syncope**

| Syncope is caused by transient global cerebral hypoperfusion either due to decreased cardiac output or excessive vasodilatation or, more commonly, a combination of both. The causes of syncope include:  **1**. **Neurally-mediated (reflex/vasovagal) syncope:** In normal circumstances physical and emotional stresses lead to increased sympathetic tone. In neurally-mediated syncope, there is initially increased sympathetic tone which is inappropriately withdrawn and replaced with increased vagal tone leading to reflex vasodilatation and/or bradycardia. This in turn leads to cerebral hypoperfusion. The physical stresses include pain, cough, micturition, or instrument playing. The classical vasovagal syncope is a neurally mediated syncope.  **2. Cardiac syncope:** Inability of the heart to maintain adequate cardiac output and thereby adequate cerebral perfusion is the pathophysiological mechanism underlying cardiac syncope. Cardiac etiology of syncope can be further subdivided into structural causes and causes due to rhythm disturbances.  **Dysrhythmias:** Both bradyarrhythmias and tachyarrhythmias can lead to syncope. Low heart rates associated with sinus node disease, Stokes-Adams attack, 2nd degree heart block, 3rd degree heart block or pacemaker malfunction lead to syncope. High heart rates associated with supraventricular or ventricular tachycardia, atrial fibrillation/flutter, ventricular fibrillation or torsades de pointes can cause syncope.  **Structural heart disease:** Structural heart diseases can cause syncope when the demands exceed the ability of the heart. Structural heart diseases that cause syncope include valvular heart diseases such as aortic stenosis, tricuspid stenosis or mitral stenosis; non-valvular conditions such as cardiomyopathy, congenital heart disease, myxoma, pericardial disease, myocardial ischemia or myocardial infarction; or extra-cardiac abnormalities such as aortic dissection, pulmonary embolism, or pulmonary artery hypertension.  **3. Orthostatic hypotension:** In normal individuals, on assuming upright posture from supine position the pooling of blood in the lower extremities causes a drop in cerebral perfusion. The carotid baroreceptors stimulate the autonomic nervous system causing increased sympathetic output and decreased parasympathetic output. Failure of the above mentioned compensatory mechanism leads to cerebral hypoperfusion and syncope. Such failure of the autonomic nervous system can be primary as seen in elderly or secondary to medications (beta blockers, calcium channel blockers), neurological disorders (diabetes, spinal cord injuries) and volume depletion due to poor intake or diuretics.  **4. Medication induced syncope:** Antihypertensives, beta blockers, calcium channel blockers, cardiac glycosides, diuretics, antiarrhythmics, antipsychotics, antiparkinsonism drugs, antidepressants, phenothiazines and nitrates can cause syncope. Substances alcohol and cocaine when used or abused can also cause syncope. The underlying mechanism responsible for syncope varies with each drug and some cause syncope by multiple mechanism. Antihypertensives, calcium channel blockers and nitrates can cause excessive vasodilation. Beta blockers blunt the autonomic response to carotid baroreceptors; beta blockers and calcium channel blockers decrease the heart’s chronotropic and ionotropic response to orthostatic stress. Diuretics can cause hypovolumeia. Cardiac glycosides and antiarrhythmics can cause syncope with their proarrhythmic properties. |
| --- |
